# Real‐time monitoring of specific oxygen uptake rates of embryonic stem cells in a microfluidic cell culture device

**DOI:** 10.1002/biot.201500479

**Published:** 2016-06-22

**Authors:** Alexandre Super, Nicolas Jaccard, Marco Paulo Cardoso Marques, Rhys Jarred Macown, Lewis Donald Griffin, Farlan Singh Veraitch, Nicolas Szita

**Affiliations:** ^1^ Department of Biochemical EngineeringUniversity College LondonLondonUnited Kingdom; ^2^ Centre for Mathematics and Physics in the Life Sciences and Experimental BiologyUniversity College LondonLondonUnited Kingdom; ^3^ Department of Computer ScienceUniversity College LondonLondonUnited Kingdom

**Keywords:** Dissolved oxygen monitoring, Microfluidic cell culture device, Quantitative cell imaging, Specific oxygen uptake rate, Stem cells

## Abstract

Oxygen plays a key role in stem cell biology as a signaling molecule and as an indicator of cell energy metabolism. Quantification of cellular oxygen kinetics, i.e. the determination of specific oxygen uptake rates (sOURs), is routinely used to understand metabolic shifts. However current methods to determine sOUR in adherent cell cultures rely on cell sampling, which impacts on cellular phenotype. We present real‐time monitoring of cell growth from phase contrast microscopy images, and of respiration using optical sensors for dissolved oxygen. Time‐course data for bulk and peri‐cellular oxygen concentrations obtained for Chinese hamster ovary (CHO) and mouse embryonic stem cell (mESCs) cultures successfully demonstrated this non‐invasive and label‐free approach. Additionally, we confirmed non‐invasive detection of cellular responses to rapidly changing culture conditions by exposing the cells to mitochondrial inhibiting and uncoupling agents. For the CHO and mESCs, sOUR values between 8 and 60 amol cell^−1^ s^−1^, and 5 and 35 amol cell^−1^ s^−1^ were obtained, respectively. These values compare favorably with literature data. The capability to monitor oxygen tensions, cell growth, and sOUR, of adherent stem cell cultures, non‐invasively and in real time, will be of significant benefit for future studies in stem cell biology and stem cell‐based therapies.

**Abbreviations**
CHO: Chinese hamster ovary
CNC: computer numerical control
DO: dissolved oxygen
FCCP: carbonyl cyanide‐4‐(trifluoromethoxy)phenylhydrazone
GUI: graphical user interface
mESC: mouse embryonic stem cells
OUR: oxygen uptake rate
PC: polycarbonate
PCC: packing‐corrected confluency
PCM: phase contrast microscopy
PDMS: poly(dimethylsiloxane)
sOUR: specific oxygen uptake rate

## 1 Introduction

The survival, proliferation and phenotype of stem cells is determined by a dynamic and complex interaction between the cellular microenvironment and the cells 1. Physico‐chemical factors, such as pH, dissolved oxygen, hydrodynamic shear stress and temperature, and biochemical factors, for example the concentrations of nutrients, metabolites and signaling factors, contribute to specific cellular fates. Among these factors, dissolved oxygen (DO) is of particular importance. Oxygen crucially acts as a signaling molecule committing cells to different lineages 2, 3, 4. Under low levels of oxygen, for example, spontaneous differentiation 5, 6, inhibition of self‐renewal 7, differentiation into cardiomyocytes 8, hematopoietic cells 9, and photoreceptors 10 were reported. Additionally, analysis of the oxygen uptake of stem cells revealed shifts in their energy metabolism. These shifts were correlated with the phenotypic changes observed in the study of inherited diseases 11, wound healing 12, and differentiation pathways 13, 14, 15, 16, 17. Therefore, the capacity to monitor the DO in a culture and to quantify the oxygen kinetics of the cells, such as the specific oxygen uptake rates (sOUR), is critical to the development of stem cell‐based therapies.

In previous work, methods to quantify specific oxygen uptake rates relied on cell sampling or cell detachment 18, 19. DO was monitored during the culture of murine hybridomas in T‐flasks equipped with optical sensors. To determine the cell densities, the cells were sampled in regular time intervals and fluorescently stained. Oxygen uptake rates and sOURs were then approximated for each time interval. In assays for drug discovery, oxygen consumption rates for a variety of cell lines and for primary cells were determined by creating temporary microchambers inside well plates equipped with optical oxygen sensors 20, 21. In these chambers of microliter volumes, oxygen diffusion is severely limited, and therefore, the depletion of oxygen within the chamber can be correlated with the oxygen consumption. To perform the assays, cells had to be re‐plated to defined cell seeding densities, and these densities were then used to calculate the sOUR. Cell sampling and cell detachment, however, have an adverse effect on the phenotype of the cells, potentially affecting the outcome of a stem cell culture. Furthermore, other key characteristics of a stem cell culture, such as cellular morphology and the spatial distribution of the cells, can only be determined by direct observation of the cell culture itself. Conversely, commercially available live cell imaging incubator systems which offer the time‐lapse imaging necessary to monitor a cell culture without disruption do not provide DO measurement 22, 23, 24.

Microfluidic cell culture devices are powerful tools to study complex and dynamic cellular microenvironments, because of the unprecedented degree of control they offer over the soluble 25, 26, and the physical 27, 28 and mechanical 29, 30, 31 microenvironment of the cells. They are excellent perfusion cell culture devices that maintain a well‐defined soluble microenvironment over the cells, finely controlling the supply of nutrients, such as oxygen 32, 33, and the level of hydrodynamic shear rates 34, 35. These devices can also be employed to create wash‐out cycles, in order to perform temporally controlled removal of auto‐ and paracrine signaling factors 36, 37. Additionally, microfluidic cell culture devices can be parallelized to increase throughput 38, 39, and they can be combined with high resolution microscopic imaging modalities for live cell imaging 38, 39, 40, 41, 42. However, when monitoring of DO for adherent cells was shown, cell enumeration was either not performed or only as an end‐point analysis 43, 44, 45, 46, 47, 48. To date, there is therefore to the best of our knowledge no system capable of monitoring cell densities and DO simultaneously, let alone in real time.

In this contribution, we show for the first time a non‐invasive and real‐time quantification of sOUR in an adherent culture system. The cells are cultured in a microfluidic device with a working volume of approximately 25 μL 34. We employ non‐invasive optical sensors and trainable image segmentation methods to analyze growth and oxygen uptake kinetics of two adherent cell lines, Chinese hamster ovary (CHO) and mouse embryonic stem cells (mESC), without any interruption or sampling of the cultures. The data obtained are consistent across independent experiments and matches sOUR values previously reported for these cell lines, yet obtained using invasive analytical techniques and with culture vessels of working volumes several orders of magnitude larger than our microfluidic culture device. The ability to monitor quantitatively, directly and in real time adherent cells in their niche provides new opportunities to further our understanding of cellular responses to the microenvironment.

## 2 Materials and methods

### 2.1 Cell culture maintenance

Mouse embryonic stem cells (mESC) were maintained as previously reported 49.

Chinese hamster ovary (CHO) K1 adherent cells (<50 passages) were grown in T‐25 flasks in Dulbecco's Modified Eagle Medium Knock‐Out (10829, Gibco, USA) supplemented with 10% v/v Fetal Bovine Serum (26140, Gibco, USA), 10% v/v Glutamax (35050, Gibco, USA) and 1% v/v Modified Eagle Non‐Essential Amino Acids (11140, Gibco, USA).

### 2.2 Microfluidic device and microfluidic cell culture

All components of the device were designed using Solidworks^®^ (Dassault Systèmes SOLIDWORKS Corp., USA) and fabricated by CNC micro‐milling (M3400E, Folken Industries, USA) as previously described 34. Briefly, two rigid polycarbonate (PC) plates formed a compressive seal over a microfluidic chip, which contained the fluidic channels and the culture chamber, against a microscope slide. The microfluidic chip was fabricated from poly(dimethylsiloxane) (PDMS); interconnects (to connect with tubing for the perfusion of culture medium) and the lid (to open and close the culture chamber) were fabricated from PC; the seal between the lid and the top PC plate was achieved by compressing a gasket made out of PDMS. In addition to the previously described design, an aluminum frame was placed around each of the two interconnects to reinforce the top plate in order to prevent liquid from leaking. The bespoke pressure‐driven pumping system consisted of a gas supply (21% O_2_, 5%CO_2_, N_2_; BOC, UK) controlled by a pressure regulator (ITV001‐2BL‐Q, SMC Pneumatics Ltd, UK) and connected to a medium reservoir (DURAN^®^ bottle with GL‐45 cap, Schott AG, Germany). The device parts, medium reservoir and tubing were sterilized by autoclaving and assembled aseptically in a biosafety cabinet. For mESC cultures, the microscope slide was coated with 0.1% w/v gelatin (G1890, Sigma‐Aldrich, UK) solubilized in Dulbecco's Phosphate Buffer Solution (D1408, Sigma‐Aldrich, UK). CHO cells were cultured without coating of the microscope slide. CHO cells were seeded in the microfluidic culture device at 3 × 10^4^ cells cm^−2^, mESCs at 5 × 10^4^ cells cm^−2^. Both cell lines were cultured under continuous medium perfusion with a flow rate of 300 µL h^−1^.

### 2.3 Respiratory assay

Carbonyl cyanide 4‐(trifluoromethoxy)phenylhydrazone (FCCP, C2920, Sigma‐Aldrich, UK) was dissolved in dimethyl sulfoxide (DMSO) at a concentration of 100 mM. It was then diluted in stem cell culture medium to obtain a final concentration of 1.5 µM. Oligomycin (75351, Sigma‐Aldrich, UK) was dissolved in 70% v/v ethanol at a concentration of 20 µg mL^−1^. It was subsequently diluted in stem cell culture medium to a final concentration of 0.2 µg mL^−1^.

Mouse embryonic stem cells were seeded in the microfluidic culture device at 5 × 10^5^ cells cm^−2^ and left to attach in the chamber in static culture conditions (no flow) overnight. The culture was perfused at 300 µL h^−1^ with a syringe drive (KDS100, KD Scientific, USA) with stem cell medium for the first 2.5 h, then stem cell medium supplemented with oligomycin for 3.5 h, and finally stem cell medium supplemented with FCCP until the end of the assay.

### 2.4 Monitoring of dissolved oxygen (DO)

Oxygen probe flow‐through cells (FTC‐PSt3, PreSens Precision Sensing GmbH, Germany) were connected to the inlet and outlet of the microfluidic culture device using standard Luer‐lock adapters (P‐660, Upchurch Scientific, USA), and read out by a transmitter (Oxy‐4 mini, PreSens Precision Sensing GmbH, Germany) via optical fibers (POF‐L2.5, PreSens Precision Sensing GmbH, Germany). Custom‐made microscope glass slides with planar oxygen sensors were supplied by PreSens Precision Sensing GmbH (Germany); the sensors were interrogated using an optical fiber brought into close proximity to the device. To align the fiber with the sensor, a bespoke collar was designed and fabricated in house. The collar was attached to the 10× microscope objective and held the optical fiber close to the objective (Supporting information, Fig. S1). Sensors were calibrated in the device prior to each experiment by supplying air‐saturated reverse osmosis water and a solution of 10 g L^−1^ of sodium sulfite (239321, Sigma‐Aldrich, UK). Phase values were monitored and calibration points were recorded when phase variations did not exceed ±0.05° over a period of 3 minutes, according the manufacturer's specifications. During culture, all three sensors (flow‐through cells at inlet and outlet, and the planar oxygen sensor) were interrogated in intervals of 15 minutes each.

### 2.5 Image acquisition and processing

Imaging of cultures in the microfluidic device was automated under a LabVIEW routine (National Instruments, USA) as previously described 42. Image acquisition occurred in intervals of 30 min. In between the image acquisition sequences, the objective was positioned such that the attached fiber optics aligned with the oxygen sensor of the cell culture device.

Image processing was carried out using our algorithms written in MATLAB^®^ (version R2014b, The MathWorks, Inc, USA). Images for a given time point were stitched together by ‘calibrating’ microscope stage positions with regard to image‐space coordinates in the culture chamber. A graphical user interface (GUI) was used to divide the stitched image (~6800 × 15 300 pixels) into small regions of interest (unless otherwise stated, a 47 × 15 grid was used). The GUI was also employed to manually de‐select the regions that did not provide information for cell growth characterization, such as the location of the oxygen sensor or uneven edges of the PDMS sidewalls of the culture chamber. Images were segmented (i.e. each pixel was classified as either 1 or 0 for ‘cell’ and ‘background’ pixels, respectively) using a recently described trainable segmentation method 50.

Confluency (area covered by cells divided by total culture area) of a given region was then computed from the segmented image as the ratio of ‘cell’ pixels to the total number of pixels. Culture‐wide confluency was computed as the mean confluency calculated from all regions.

Cell density (e.g. number of cells, *X*, per unit area) was calculated from the confluency values using the trainable segmentation method 50 and the packing‐corrected confluency (PCC) 49.

### 2.6 Numerical and statistical analysis

Growth rates, μ, were calculated using

(1) $$\mu =\frac{\ln\left(X\text{/}X_{\text{0}}\right)}{t}$$

where *X*_0_ is the initial number of cells and *X* the number of cells at a time *t*.

Doubling times, *g*, were calculated using

(2) $$g=\frac{\ln\left(\text{2}\right)}{\mu }$$

Specific oxygen uptake rates (sOUR) were calculated similar to Mehta et al. 51 using

(3) $$\mathrm{sOUR}=\frac{Q(O_{\text{2},\mathrm{in}}-O_{\text{2},\mathrm{out}})}{X}$$

where *Q* is the volumetric flow rate, *O*_2,__*in*_ and *O*_2,__*out*_ are the concentrations of the oxygen at the inlet and outlet, respectively.

Standard deviations, σ, were calculated using

(4) $$\sigma =\sqrt{\frac{\sum^{\text{​}}{\left(x-\bar{x}\right)}^{\text{2}}}{n}}$$

where *x* is the replicate value, *x* the sample mean and the sample size.

## 3 Results

### 3.1 Non‐invasive multi‐modal monitoring of cell cultures in the microfluidic cell culture device

**Figure 1:**
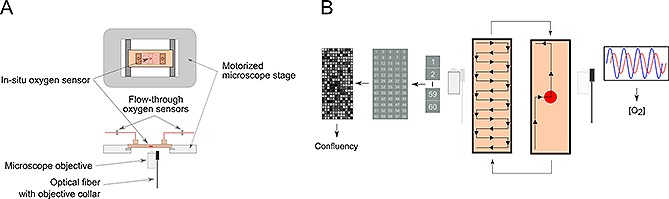
Experimental setup for the real‐time monitoring of cell growth and dissolved oxygen (DO) in a microfluidic cell culture device. (**A**) Schematic representation of the microfluidic device placed on a motorized stage of an inverted microscope; two oxygen flow‐through sensors are used to monitor the perfused culture medium (inlet) and the spent medium (outlet); a bespoke collar held the optical fiber, used for the interrogation of the in‐situ oxygen sensor, in place. (**B**) Schematic representation of the automation of image acquisition and interrogation of the in‐situ oxygen sensor.

The microfluidic cell culture device was placed on a motorized stage of an inverted fluorescence microscope for non‐invasive monitoring. To perform the stem cell culture, the microscope and the pressure‐driven pump were automated under a LabVIEW routine. Monitoring of cell culture growth was carried out by the periodic acquisition and subsequent processing of phase contrast microscopy (PCM) images. Dissolved oxygen (DO) was monitored at three locations (Fig. 1A): upstream and downstream of the culture chamber by positioning oxygen flow‐through probes at the inlet and outlet of the culture device, respectively; and in situ, by placing an oxygen sensor in the center of the bottom of the culture chamber. A bespoke collar attached to the 10× microscope objective (Supporting information, Fig. S1) enabled to interchangeably acquire PCM images (via the objective) and read out the in situ oxygen sensor. The LabVIEW routine controlled the automated acquisition of the set of images required to monitor the growth of the stem cell culture within the culture chamber (Fig. 1B). In order to minimize the time during which the cells were exposed to high intensity white light illumination, the acquisition sequence was executed in intervals of 30 minutes only.

### 3.2 Cell expansion in the microfluidic cell culture device

**Figure 2:**
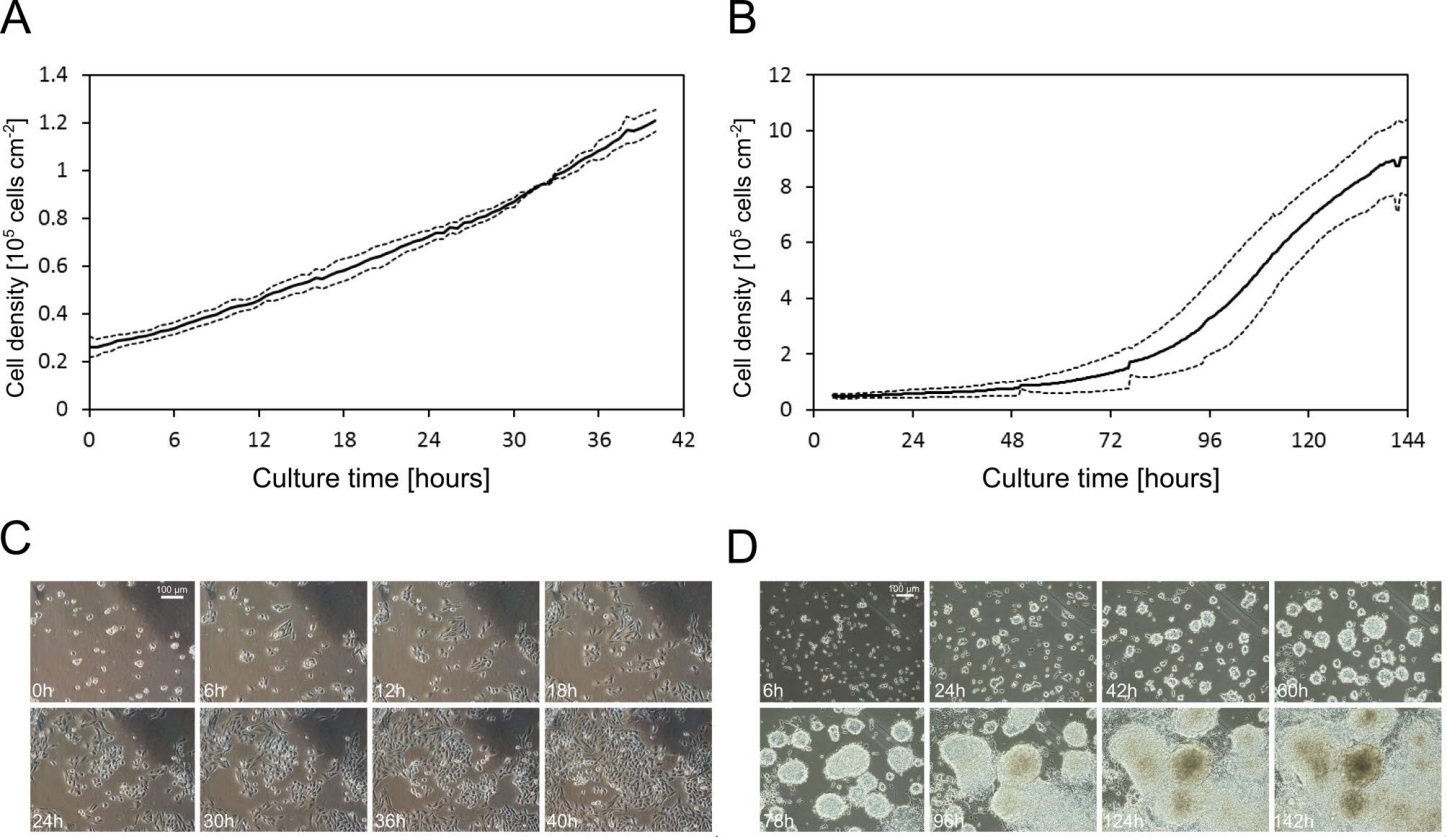
Imaging‐based monitoring of cell expansion in the microfluidic cell culture device. (**A**) Time‐course data of cell densities obtained from phase contrast microscopy (PCM) images for CHO cultures (solid and dashed lines represent the mean, *n* = 3, and one standard deviation). (**B**) Time‐course data of cell densities obtained from PCM images for mESC cultures (solid and dashed lines represent the mean, *n* = 2, and one standard deviation). (**C**) PCM images (10× magnification) of a selected field of view of a CHO culture at specific time points.(**D**) PCM images (10× magnification) of a selected field of view of a mESC culture at specific time points.

To validate the multi‐modal monitoring, continuous cultures of Chinese hamster ovary cells (CHO) were performed. During each image acquisition sequence, the entire culture chamber was scanned. The image‐processing algorithm generated an average cell density value from 507 image regions covering the culture chamber (198 regions were discarded from the analysis) within minutes. Given that the interval between acquisitions was 30 minutes, this approach offered the online monitoring of cell growth and is thus suitable for decision‐making and the early detection of anomalies, i.e. deviations from a known or expected growth pattern. A growth curve averaged from three independent CHO cells cultures in the microfluidic device is presented in Fig. 2A. No lag phase was observed in any of the cultures. Cell densities exceeded 1 × 10^5^ cells cm^−2^ after 40 h and final confluency values exceeded 75% (Fig. 2C). The calculated maximal growth rate (µ_max_) for CHO cells was 0.041 ± 0.006 h^−1^, which corresponded to a doubling time (*g*) of 17.1 ± 2.2 h. The reproducibility between cultures was good with less than 5% variation on average. Mouse embryonic stem cells (mESC) were perfused for six days (144 h) with the same flow rate as the CHO cells. The averaged growth curve is presented in Fig. 2B. mESC cultures exhibited a long lag phase (72 h), reached final cell densities of 9 × 10^5^ cells cm^−2^, and attained confluency values larger than 80% (Fig. 2D). The µ_max_ and *g* for mESC were 0.035 ± 0.004 h^−1^ and 19.9 ± 1.9 h, respectively. The reproducibility with mESC was lower than for CHO cells, with approximately 30% variation on average between cultures. The growth profile of mESC in the microfluidic cell culture device was comparable to those observed in static T‐flask cultures (Supporting information, Fig. S2).

### 3.3 Monitoring of dissolved oxygen and detection of respiratory events

**Figure 3:**
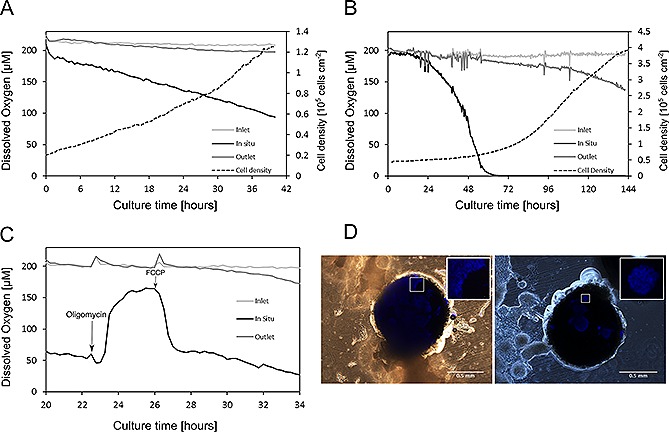
Monitoring of dissolved oxygen (DO) concentrations. (**A**) DO profiles for a typical CHO culture in the microfluidic device. The corresponding growth curve (dashed line) is also shown for reference. (**B**) DO profiles and corresponding growth curve for a typical mESC culture in the microfluidic device. (**C**) DO profiles for the respiratory assay with mESCs in the microfluidic device. Oligomycin is injected after 2.5 h of perfusion culture, FCCP after 6 h as indicated by the labeled arrows. (**D**) Composite microscopy images (phase contrast/epifluorescence, 4× magnification) of the in‐situ sensor taken from two independent culture experiments. Cells overgrowing the sensor appear in blue as they stain positively for DAPI. Insets provide enlarged views of the DAPI‐stained nuclei for each experiment.

Dissolved oxygen (DO) of the CHO culture was monitored in the microfluidic device (Fig. 3A). As expected, the signal from the flow‐through sensor placed at the inlet was stable throughout the culture experiment. The values of DO at the outlet steadily decreased from ~215 µM at the start of the culture to ~195 µM after 40 h of culture.

In‐situ oxygen levels were monitored using the sensor located in the center of the culture chamber. In contrast to the slow decrease of the oxygen concentrations measured at the outlet, the in‐situ oxygen rapidly decreased, reaching ~95 µM at the end of the culture.

DO monitoring of a six‐day mESC culture presented profiles similar to the CHO culture (Fig. 3B): inlet measurements were stable throughout the culture; outlet measurements showed a steady decrease from ~200 µM at the start of the culture to ~140 µM after six days. Infrequent spikes of the DO values were observed at the inlet and outlet sensor at the same time. The in‐situ sensor recorded a total depletion of oxygen already within 72 h of culture (at which point the cell density was 9 × 10^5^ cells cm^−2^).

To assess whether rapidly changing culture conditions can be detected, a respiratory assay was conducted with mESC (Fig. 3C). Cells were seeded in the culture device, cultured overnight in static conditions (no flow) and subsequently exposed for 2.5 h to an ATP synthase inhibitor, oligomycin, and then to a respiratory uncoupler, FCCP. Spikes in the signal of the sensors at the in‐ and outlet were observed when the culture medium was switched; first from basal stem cell culture medium to oligomycin‐supplemented medium, and then from oligomycin‐supplemented medium to FCCP‐supplemented medium. In between these changes, the signal from the flow‐through sensor at the inlet was stable. The sensor at the outlet showed that oxygen levels started to decrease after 9 h, from ~200 µM to ~180 µM, i.e. long after the addition of FCCP. In‐situ oxygen levels decreased steadily from ~65 µM to ~55 µM in the 2.5 h preceding the addition of oligomycin. After the addition, oxygenvalues increased rapidly to reach a plateau of ~165 µM. Once FCCP had been introduced, oxygen values dropped abruptly to ~65 µM in less than 2 h. Following this steep decrease, oxygen concentrations then decreased only slowly, and a concentration of ~35 µM was recorded after 13 h of culture.

Cells' nuclei were stained with DAPI at the end of the culture (Fig. 3D) to assess if any cell overgrowth occurred on top of the in‐situ oxygen sensor. Stained cells were observed on the surface of the sensor in all experiments.

### 3.4 Quantification of oxygen consumption and specific oxygen uptake rate

**Figure 4:**
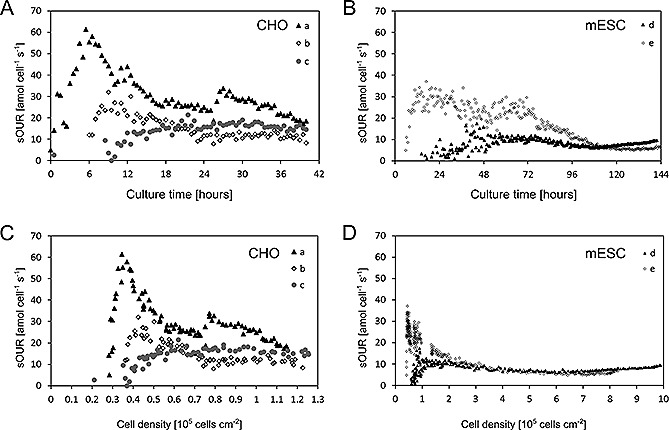
Non‐invasive and real time monitoring of specific oxygen uptake rates (sOURs) of CHO and mESC cultures. (**A**) Time‐course data of sOURs for three independent replicate CHO cultures (a, b and c). (**B**) Time‐course data of sOURs for two independent replicate mESC cultures (d and e). (**C**) and (**D**) sOUR data of CHO and mESC cultures, respectively, plotted against cell densities. Negative sOUR data obtained during the early stages are not shown. Negative values were obtained in some of the cultures and were due to instrument noise, i.e. the noise was of higher amplitude than the difference between the oxygen measurement at the inlet and outlet.

The oxygen consumption was computed as the difference between the DO measured at the inlet and at the outlet and divided by the number of cells to obtain the specific oxygen uptake rate (sOUR). Time‐course sOUR data were obtained for CHO cells for independent culture experiments (Fig. 4A). When plotted against cell density (Fig. 4C), the sOUR for the CHO cells was found to initially increase with cell density, before reaching stable values of ~20 amol cell^−1^ s^−1^ on average for cell densities greater than 5 × 10^4^ cells cm^−2^. A maximal sOUR value of 60 amol cell^−1^ s^−1 ^was recorded at lower cell density (3.5 × 10^4^ cells cm^−2^). Time‐course sOUR data obtained from independent mESC cultures presented similar trends to the CHO cultures (Fig. 4B): sOUR increased with cell density, until a cell density of 10^5^ cells cm^−2^, and then plateau‐ed at values of ~10 amol cell^−1^ s^−1^ on average for cell densities greater than 5 × 10^5^ cells cm^−2 ^(Fig. 4D). The maximal sOUR value measured was 35 amol cell^−1 ^s^−1^ at 5 × 10^4^ cells cm^−2^.

## 4 Discussion

### 4.1 Oxygen monitoring in the microfluidic cell culture device

We described the advances made to achieve real‐time monitoring of dissolved oxygen and cell density, which allowed real‐time quantification of specific oxygen uptake rates (sOUR). Non‐invasive monitoring of dissolved oxygen (DO) was successfully demonstrated. The optical sensors have a detection range between 0 and 45 mg L^−1^ of DO, which clearly extends beyond the reported maximal solubility of oxygen in cell culture media at 37°C 52. Culturing cells under continuous perfusion allowed the determination of the OUR by measuring DO levels at the inlet and outlet. Spikes in the signals of the inlet and outlet sensors were occasionally observed. However, these occurred at identical time points for the inlet and outlet sensor, and thus did not affect the differential measurement employed to obtain the value for the oxygen consumption.

There was no obvious correlation between the bulk oxygen consumption rate and the apparent peri‐cellular oxygen levels measured by the in‐situ oxygen sensor. This requires further investigation, but a possible explanation for the rapid depletion of oxygen measured in‐situ could be the growth of cells on top of the sensor. End‐point immunofluorescence assays showed that cells were indeed able to proliferate on the sensor, possibly due to the gelatin coating applied prior to the cell seeding (Fig. 3D). With cells consuming the oxygen at the sensor surface, the measured values might no longer be representative of the peri‐cellular oxygen levels (i.e. as experienced by the cells). Moreover, the cells themselves might physically limit the transport of oxygen molecules from the surrounding bulk liquid to the sensor surface 53. To improve the in‐situ oxygen measurement, it would therefore be necessary to inhibit cell attachment via surface modification of the sensor 54, 55, 56.

### 4.2 Detection of respiratory events

We exposed the mESC first to oligomycin, inhibiting mitochondrial respiration by preventing the proton movement through the F_1_F_0_ ATP synthase, and then to FCCP, a mitochondrial uncoupler that stimulates electron transport and oxygen uptake 17. The characteristic decrease in oxygen uptake by cells that undergo oligomycin‐inhibited cellular respiration was observed in the hour following the addition of the compound to the culture medium. FCCP‐stimulated respiration was measured within minutes following the addition of FCCP. This demonstrates that the in‐situ sensor is capable of detecting changes in the respiration of cells as induced by changing culture conditions, in spite of cell overgrowth. Furthermore, this experiment also highlighted that fast transient cellular responses can be detected. These would be harder to detect from the outlet sensor; the comparatively low flow rates would impose a time delay (from the onset of the respiratory change until the affected culture medium volume increment reaches the outlet sensor) which would be prohibitive to detect rapid changes.

### 4.3 Specific oxygen uptake rate monitoring

Specific oxygen uptake rates were determined from cell densities, calculated with our packing‐corrected confluency (PCC – a number that takes into account the variations in the degree of the packing of cell clusters as described and validated in Jaccard et al. 49) and a trainable segmentation algorithm, and from the oxygen consumption which was computed from the difference of the oxygen values recorded at the inlet and outlet sensor. Our image analysis method proved robust enough to enable the detection of the cells during an entire culture, i.e. from the individual round cells shortly after cell seeding, to the packed cell layers typical for higher cell densities.

Negative sOURs obtained at the early stages of some of the cultures were due to instrument noise, i.e. the noise was of higher amplitude than the difference between inlet and outlet measurements (The negative values were omitted in the graphs of Fig. 4, as they are solely measurement artifacts without any physical or biological meaning).

### 4.4 Growth and oxygen kinetics in the microfluidic cell culture device

For both CHO and mESC cultures, the sOUR increased with cell density in the early phase before reaching stable values. For CHO cells, the sOURs were between 8 and 35 amol cell^−1^ s^−1^, and the highest measured sOUR was 60 amol cell^−1^ s^−1^ (Fig. 4C). These values are well within the range reported in the literature for anchorage‐dependent CHO cultures (Table 1).

**Table 1:** Literature values for specific oxygen uptake rates (sOUR) for different mammalian and stem cell lines.

Cell type	Culture type	Measured sOUR (amol cell^‐1^ s^‐1^)	Reference
H460	Adherent	30	62
A549	Adherent	25	62
CHO	Adherent	94	63
	Suspension	139	59
		8	57
		43	64
		35	65
		16–64	60
		56	66
		50–89	67
		46–67	68
		86	69
Fibroblast	Adherent	60	70
Hepatocyte	Adherent	240–330	70
hESC	Adherent	110–112	71
		1–4	15
		1	72
hMSC	Adherent	0.5–3	73
Hybridoma	Adherent	49	74
	Suspension	43–68	60
mESC	Adherent	10–29	6
		28	75
		25–33	76
	Spheroids	40	77
mNSC	Spheroids	31	78

Maximal growth rate (µ_max_) values computed from the growth curves (Fig. 2A) also matched with values previously reported in literature 57. For mESC, steady state sOURs were between 5 and 15 amol cell^−1^ s^−1^, and the highest measured was 35 amol cell^1^ s^−1^ (Fig. 4D). The sOUR and µ_max_ values again compare favorably with the range of values reported in literature, i.e. in Table 1, and in Tamm et al. 58, respectively. With both cell lines, sOURs followed a similar trend over time: early increase with cell density, with in some of the cultures a spike in the sOUR, followed by a decrease of the values until a subsequent plateau is reached until the end of the culture. The larger sOUR values early in a culture have also been presented in previous studies, performed in batch and continuous suspension culture systems 59, 60: cells undergo an adaptation phase at the beginning of a culture during which sOUR is at its highest. It will then decrease to reach a steady state. In the case of the mESC, the period during which the sOUR fluctuates overlaps with the growth lag phase (Fig. 4B). As CHO cells did not exhibit any growth lag phase, we are not able to make the same correlation. In order to understand fully what causes these sOURs variations over time, it would be necessary to analyze other inputs and outputs of the cellular metabolism such as glucose, lactate, dissolved CO_2_ and ATP. However this is beyond the scope of this work. A potential limitation of this approach concerns the estimation of cell density based on PCC, which makes the assumption that the cells grow in a monolayer 49. While this assumption holds true for CHO cells and for a large number of adherent cell lines, it does not for dense, overgrown, and 3D‐like colonies as observed at the end of the mESC cultures.

### 4.5 Conclusions

In summary, we successfully demonstrated the real‐time monitoring of cell growth and respiration for two cell lines, CHO and mESCs in a microfluidic device. Time‐course data for cell density and dissolved oxygen obtained from an adherent cell culture represents a degree of monitoring which has thus far only been known from suspension culture systems 61. Furthermore, these were obtained in culture vessels with operating volumes several orders of magnitude higher than the volume of the culture chamber in our microfluidic device. It should be emphasized that by using phase contrast images for the analysis of cell growth, it was not necessary to label the cells or sample the culture, and that therefore the measurement of the sOUR is truly non‐invasive. Finally, the advances in monitoring presented here will also enhance the applicability of microfluidic devices for stem cells. The capacity of these devices to generate well‐defined and dynamic culture conditions is well documented. By combining these advantages with label‐free and real‐time monitoring of oxygen concentrations and cell proliferation, it will become possible to detect metabolic shifts and oxygen response of the cells without interfering with their integrity or that of the culture.

## Supporting information

As a service to our authors and readers, this journal provides supporting information supplied by the authors. Such materials are peer reviewed and may be re‐organized for online delivery, but are not copy‐edited or typeset. Technical support issues arising from supporting information (other than missing files) should be addressed to the authors.

Supporting Information
