# Supplementary material for: Real‐time monitoring of specific oxygen uptake rates of embryonic stem cells in a microfluidic cell culture device
Source: Biotechnol J. 2016 Jun 22;11(9):1179–89. doi: 10.1002/biot.201500479 (PMC5103178; doi:10.1002/biot.201500479)
Supplement: Supplementary file 1 — Supporting Information [file BIOT-11-1179-s001.pdf]

Biotechnology
Journal

Supporting Information for DOI 10.1002/biot.201500479

Real-time monitoring of specific oxygen uptake rates of embryonic stem cells in a microfluidic cell culture device

Alexandre Super, Nicolas Jaccard, Marco Paulo Cardoso Marques, Rhys Jarred Macown, Lewis Donald Griffin, Farlan Singh Veraitch, Nicolas Szita

© 2016 Wiley-VCH Verlag GmbH & Co. KGaA, Weinheim

Supporting Information – Super et al., Real-time monitoring of specific oxygen uptake rates of embryonic stem cells in a microfluidic cell culture device.

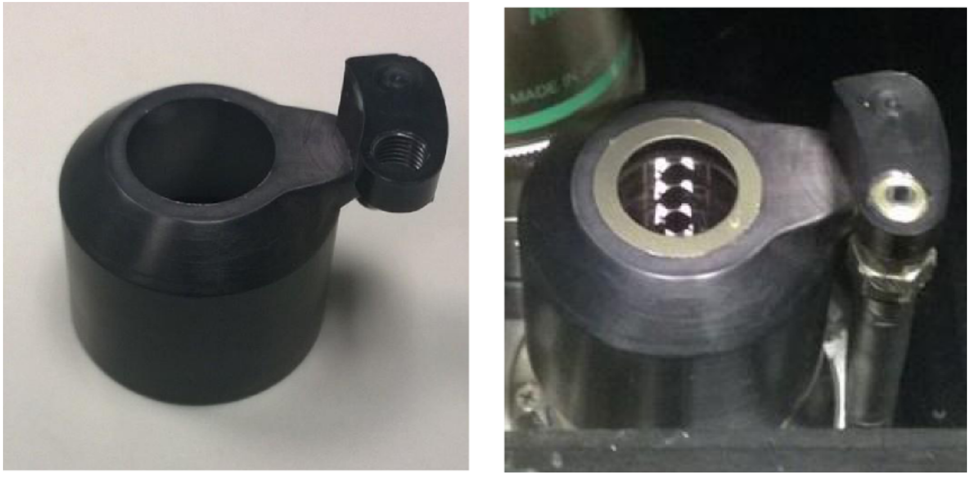

Supporting Information, Figure S1: Photograph of the bespoke collar (left). Bespoke collar attached to a 10x microscope objective with an optical fiber (right).

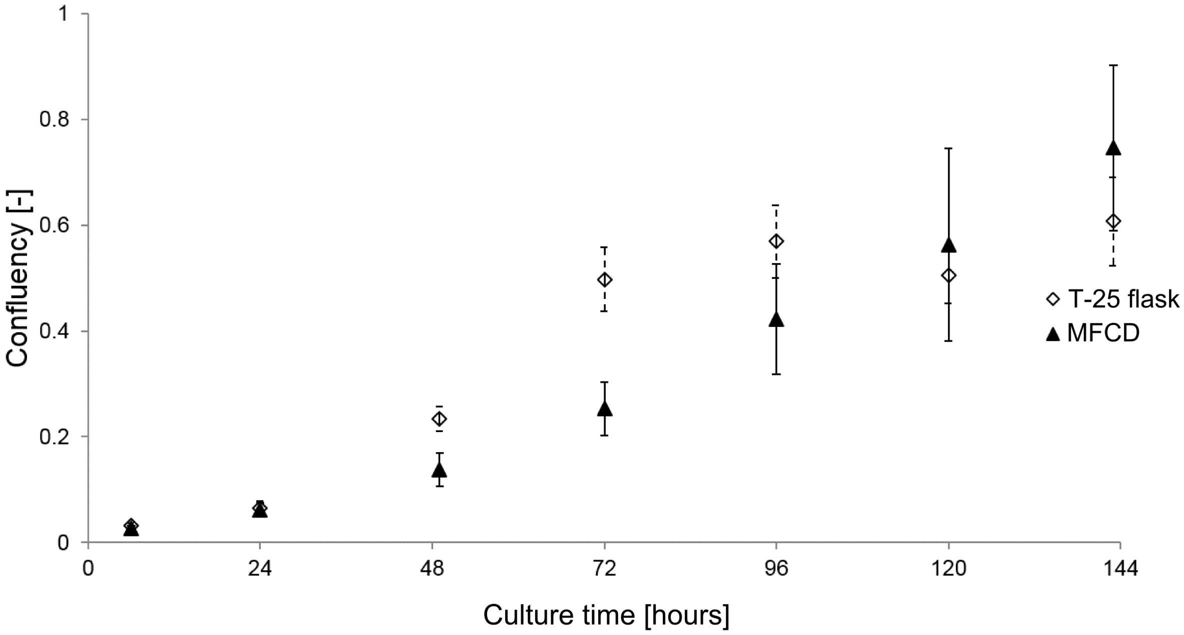

Supporting Information, Figure S2: Growth curves of mouse embryonic stem cells (mESCs) grown in a T-25 flask (open symbol) and in the microfluidic culture device (MFCD, closed symbol). Each point represents the average computed confluency of ten 10x

magnification PCM images. The error bars represent the standard variation of the average confluency.
